# Isotopic analysis of island House Martins *Delichon urbica* indicates marine provenance of nutrients

**DOI:** 10.1111/ibi.12150

**Published:** 2014-03-27

**Authors:** Adam D P Cross, Jonas Hentati-Sundberg, Henrik Österblom, Rona A R McGill, Robert W Furness

**Affiliations:** 1 College of Medical, Veterinary and Life Sciences, University of GlasgowGlasgow, UK; 2 Stockholm Resilience Centre, Stockholm UniversityStockholm, Sweden; 3 Scottish Universities Environmental Research CentreEast Kilbride, UK

**Keywords:** Common Guillemot, food webs, passerines, stable isotope analysis

## Abstract

The presence of one of the largest colonies of House Martins in Europe on the small island of Stora Karlsö, Sweden, led us to investigate the source of their food by analysis of stable isotopes of carbon and nitrogen. Carbon isotopic values of House Martin nestlings were the same as those of Common Guillemot *Uria aalge* nestlings fed on marine fish, but differed from local Collared Flycatcher *Ficedula albicollis* nestlings fed on woodland insects. We infer that these House Martins fed their chicks almost exclusively on insects that had used nutrients derived from seabirds, indicating a dependence on the presence of a large seabird colony. We suggest by extension that some populations of island passerines of high conservation importance may also be dependent on nutrient subsidies from seabird colonies.

Trophic cascades have been documented for a wide range of terrestrial and marine ecosystems, resulting from a growing understanding of energy flows and species interactions within systems (Pace *et al*. 1999). Such studies can explain factors influencing the structure and function of ecosystems and contribute towards an increased understanding of the role of human actions in shaping such dynamics (Shurin *et al*. 2002, Nyström *et al*. 2012). Recent studies of trophic cascades across ecosystems (Knight *et al*. 2005) highlight a number of issues associated with complex management of cross-scale dynamics (Cash *et al*. 2006). In particular, the effect of human actions on aquatic systems may be more significant than on terrestrial systems (Shurin *et al*. 2002). This sensitivity in aquatic systems may result in significant changes to consumer populations if perturbed. This disturbance could then propagate throughout aquatic environments and also influence terrestrial systems, which are subsidized by aquatic trans-boundary input (Kolb *et al*. 2010b). The trans-boundary input of nutrients is an increasingly recognized example of such cross-scale dynamics (Layman *et al*. 2012), which can have significant impacts upon recipient communities (Ellis 2005, Young *et al*. 2010, Caut *et al*. 2012).

In this study, we focus on seabirds as an important link in cross-scale dynamics. Seabirds have been shown to influence insular systems where they breed (Mulder *et al*. 2011, Caut *et al*. 2012) by bringing onto land large quantities of nutrients through prey remains, eggs, feathers, carcasses and especially the deposition of guano (Siegfried *et al*. 1978). Nitrogen-rich guano frequently influences terrestrial systems (Ellis 2005), but it can also influence surrounding coastal waters through nutrient run-off (Bosman & Hockey 1986, Kolb *et al*. 2010a,b). These marine subsidies may subsequently influence coastal communities, resulting in elevated nutrient levels, algal production and insect density (Bosman & Hockey 1986, Kolb *et al*. 2010a). Consequently, these subsidies may again feed back onto islands by terrestrial consumers feeding upon organisms that are themselves supported by nutrient run-off from seabird colonies.

We specifically investigated nitrogen and carbon stable isotopes, a commonly used tracer of nutrient transfer between food webs (Inger & Bearhop 2008), and focused on feathers in terrestrial House Martin *Delichon urbica* nestlings to trace seabird-derived nutrients. An individual's dietary selection can be inferred from the isotope signal of the feathers for the period over which they were grown and irrigated with blood (Forero & Hobson 2003, Pearson *et al*. 2003). Nitrogen isotopes indicate the trophic level at which animals were feeding, while isotopes of carbon differ in relative abundance between marine and terrestrial/freshwater ecosystems and thus indicate the source of carbon (Inger & Bearhop 2008). Breeding House Martins typically feed on flying terrestrial or freshwater insects within about 0.75 km of the nest, with an average foraging range of 0.45 km (Bryant & Turner 1987, Forrester & Andrews 2007). The abundant insect community often associated with seabird colonies (Sanchez-Piñero & Polis 2000, Kolb *et al*. 2010a) potentially represents a large, marine-derived prey source for such aerial insectivorous passerines. This study focuses upon an unusually large and expanding House Martin colony situated above a large and also expanding seabird colony on a small island in the Baltic Sea. To our knowledge, there are no published examples of nutrient transfer from seabirds to passerines. We thus test the hypothesis that House Martins on Stora Karlsö are strongly associated with and dependent upon changes in the Baltic marine food web, mediated through ornithogenic insect prey rather than terrestrial autochthonous insects.

## Methods

**Figure 1:**
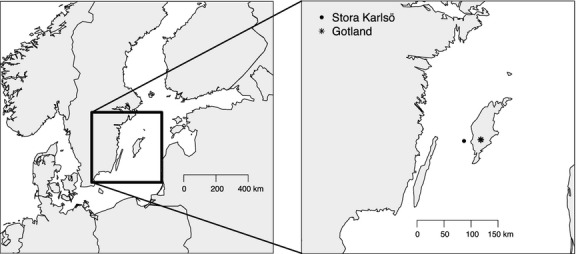
Location of the sampling sites in the Baltic Sea.

The Swedish island of Stora Karlsö (57^o^17′N, 17^o^58′E) in the Baltic Sea, *c*. 6.5 km off the west coast of Gotland (Fig.1), holds most of the breeding Common Guillemot *Uria aalge* population in the Baltic, with about 10 000 pairs nesting on limestone cliffs on the east coast, as well as large numbers of Razorbills *Alca torda* (Kadin *et al*. 2012). A lighthouse situated on top of the main seabird cliff provides nest-sites for a large colony of House Martins, numbering 150 pairs in 2013. To determine the extent to which seabirds influence the diet of terrestrial passerines, feathers were collected from nestlings of Common Guillemots, House Martins and Collared Flycatchers *Ficedula albicollis*. Feathers were analysed for the stable isotope ratios of nitrogen and carbon. Common Guillemot nestlings are fed entirely on small fish from the Baltic Sea (Kadin *et al*. 2012), thus representing an isotopic endpoint for a marine diet. Collared Flycatcher nestlings in the population sampled are fed on woodland caterpillars (Veen *et al*. 2010), representing the isotopic endpoint for the terrestrial diet. Variation in isotope values of carbon and nitrogen of House Martin nestling feathers, relative to Collared Flycatcher and Common Guillemot feathers, could thus be attributed to the indirect influence of ornithogenic prey.

Single feathers (tertials) were collected from one House Martin nestling from each of 16 nests on the lighthouse at Stora Karlsö between 30 June and 5 July 2013. Single feathers (primary coverts) were collected from each of 15 Common Guillemot nestlings captured below the cliff at Stora Karlsö as they fledged on 30 June 2013. Single feathers (tertials) were collected from one Collared Flycatcher nestling from each of 23 nestboxes (30 km from the House Martin colony) on Gotland from 23 to 25 June 2013. Cleaning or preservation agents may alter isotopic ratios (Quillfeldt *et al*. 2010). Consequently, feathers were collected, visually inspected for contamination (only clean feathers were retained) and stored dry prior to analysis (Michalik *et al*. 2012). In the laboratory, feather barbs from a sample were cut from the rachis with sterilized scissors and weighed precisely (*c*. 0.7 mg) in individual tin cups for isotopic analysis. Carbon and nitrogen isotopes were analysed by continuous flow isotope ratio mass spectrometry (CF-IRMS) with a Costech ECS 4010 elemental analyser linked to a Thermo Scientific Delta V mass spectrometer. The stable isotope ratios were expressed in δ values as parts per thousand (‰). Internal standards are traceable to the following international standards, AIR for nitrogen and PeeDee Belemnite for carbon. Instrumental drift was corrected by means of the repeated measurement of two laboratory standards every 10 samples (alternating between gelatine and two isotopically distinctive alanines). Errors were small with standard deviations less than 0.04% for carbon and 0.15% for nitrogen, based on repeated measurements of laboratory tryptophan.

## Results

**Figure 2:**
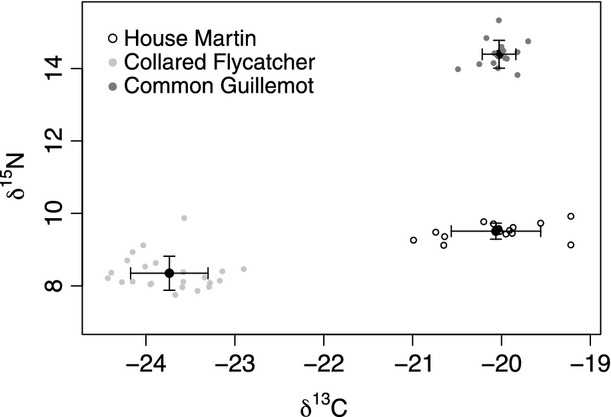
Stable isotope values in solid black symbols (‰, mean ± 1 sd) for bird feathers. Scattered points around the means represent individual samples for each species.

Feathers analysed for δ^15^N and δ^13^C exhibited a range of isotopic values between species and individuals (Fig.2). Mean values ± 1 se (and range) of δ^15^N for each species were: House Martin = 9.51 ± 0.06 (9.12–9.92), Collared Flycatcher = 8.35 ± 0.10 (7.75–9.87) and Common Guillemot = 14.39 ± 0.10 (13.82–15.33). Mean values ± 1 se (and range) of δ^13^C for each species were: House Martin = −20.06 ± 0.13 (−20.99 to −19.22), Collared Flycatcher = −23.74 ± 0.09 (−24.43 to −22.90) and Common Guillemot = −20.03 ± 0.05 (−20.49 to −19.70). There was a significant segregation in isotope values of feathers between species (multivariate analysis of variance (MANOVA): Wilks' lambda = 0.002, *F*_2,51_ = 571.6, *P *<* *0.001; Fig.2) and both nitrogen and carbon isotopes contributed significantly to the difference between species (analysis of variance (ANOVA): δ^15^N, *F*_2,51_ = 541.4, *P *<* *0.001; δ^13^C, *F*_2,51_ = 1155.9, *P *<* *0.001). *Post hoc* Tukey tests showed a significant difference in δ^15^N between all three species (*P *<* *0.001). The *post hoc* tests for δ^13^C showed that, although there were significant differences between the pairs Collared Flycatcher–Common Guillemot and Collared Flycatcher–House Martin (*P *<* *0.001), the difference between House Martin and Common Guillemot was not significant (*P *=* *0.967). When comparing the difference in mean isotopic values between species, the largest differences were seen for nitrogen (6.04‰ between Collared Flycatcher and Common Guillemot, Table1). Concomitant with the Tukey test results, the smallest observed difference was −0.04‰, between the carbon isotope samples of House Martin and Common Guillemot.

**Table 1:** The difference in isotope values between pairs of species

Species comparison	Δ δ^15^N (‰)	Δ δ^13^C (‰)
Collared Flycatcher–Common Guillemot	6.04 (5.73, 6.35)	3.71 (3.38, 4.04)
Collared Flycatcher–House Martin	1.16 (0.86, 1.47)	3.67 (3.35, 3.99)
House Martin–Common Guillemot	4.88 (4.55, 5.22)	−0.04 (−0.39, 0.32)

Values in parentheses are lower and upper 95% confidence intervals.

## Discussion

We observed significant segregation in both nitrogen and carbon isotope values between the feathers of the three bird species (Fig.2). In particular, the carbon isotopic value of the ‘terrestrial' House Martin was almost identical to that of the marine Common Guillemot, indicating a clear dependence of the terrestrial bird species on marine nutrients. The segregation of each species' feathers by its isotope values is related to δ^13^C and δ^15^N variation in the diet; Common Guillemots feed predominantly on Sprats *Sprattus sprattus* (Kadin *et al*. 2012), Collared Flycatchers feed on a terrestrial-based (woodland) insect population, and House Martins feed on an insect population, for which the carbon isotope value in chick feathers indicates is essentially entirely subsidized by marine nutrients, most likely made available through seabirds.

The link between the observed nitrogen and carbon isotope values of House Martins and Common Guillemots is most likely through a marine prey source. It is most probable that the insect population that House Martins feed upon comprises adult Chironomidae (H. Elmqvist and Y. Brodén, pers. comm.). Chironomidae emerge from coastal environments in the Baltic Sea where they are often found at much higher densities in proximity to seabird colonies (Kolb *et al*. 2010a). Chironomidae larvae near to seabird colonies are also found to have enriched δ^15^N values, reflecting their potential use of ornithogenic nutrients, which have been transported to coastal environments (Kolb *et al*. 2010b, 2012). Adult Chironomidae live for not more than a few days on average and do not feed extensively (Armitage *et al*. 1995), which would result in their stable isotope values reflecting that of the seabird-derived nutrients assimilated during larval development.

There are, to our knowledge, no previous examples of ornithogenic allochthonous input influencing insectivorous passerines, and relatively few examples of how nutrients can be traced from trans-boundary input into insectivorous passerines. Examples of allochthonous input traced in passerines include Tree Swallows *Tachycineta bicolor* in western Canada containing sewage-derived nitrogen as a result of feeding on emergent aquatic insects influenced by riverine sewage input (Wayland & Hobson 2001), and Eurasian Wrens *Troglodytes troglodytes* enriched in δ^15^N as a result of feeding on invertebrates, which in turn feed on salmon carcasses in North America (Christie *et al*. 2008). Furthermore, *Cinclodes* spp. of South America exhibit marine signatures when strongly associated with foraging in coastal environments compared with inland species of the same genus (Sabat & del Rio 2002). In contrast, isotopic signatures of different insectivorous passerine species, when uninfluenced by marine or anthropogenic inputs, appear to be consistent in their stable isotope values, in particular for δ^13^C (Hobson 1999). This consistency of stable isotope values between species also supports the use of a different insectivorous passerine species as a control sample in our study.

There is the possibility that observed differences in carbon values between the two insectivorous species could be attributed to other factors influencing carbon isotope ratios besides House Martins feeding on ornithogenic insects; however, these factors are considered unlikely given the difference in magnitude. For example, δ^13^C values vary between plants with different photosynthetic pathways, i.e. C3, C4 or CAM. Given that plant species on both Stora Karlsö and Gotland are predominantly C3 plants, the enrichment of ^13^C observed in C4 or CAM plants is unlikely to contribute to the enriched signature of House Martins from herbivorous insect prey sources (Rubenstein & Hobson 2004). Both Stora Karlsö and Gotland are also at similar altitudes and latitudes and thus the effect of these factors on carbon isotopic ratios will not influence the results. The use of sulphur isotopes (^34^S/^32^S) in identifying the origin of the House Martin prey is another potential tool to determine whether nutrients are derived from a marine or a terrestrial source (Hobson *et al*. 1999). However, for this study the feathers sampled were not of sufficient mass to be analysed for both sulphur and nitrogen and carbon isotopic analysis.

The significant increase in ^15^N between Collared Flycatcher and House Martin also suggests the latter are feeding on more enriched ^15^N prey, attributed to the input of seabird nutrients. However, the δ^15^N values of House Martins are considerably lower than those of Common Guillemot chicks. This is not surprising, firstly as seabirds excrete nitrogen that is depleted relative to the ingested food (Bird *et al*. 2008), and secondly, as nutrient run-off into coastal waters would probably result in a lowering of the δ^15^N values due to dilution during transportation and within the Baltic Sea. This in turn would result in less enriched δ^15^N values of Chironomidae larvae and thus House Martins, relative to Common Guillemots.

An alternative prey source for House Martins may be terrestrial arthropods, which are often found in high abundance when feeding upon ornithogenic detritus (Polis & Hurd 1995, 1996, Sanchez-Piñero & Polis 2000). Bird *et al*. (2008) inferred that the nitrogen signature of ammonia was especially depleted, as the guano nitrogen signature was not dramatically different from that of the birds' food. This suggests that the nitrogen taken up by insects feeding within the seabird colony could be derived from ammonia rather than from the uric acid component of excreta.

House Martins breed throughout much of Europe, but their colonies are typically of fewer than five pairs and only about 1% of colonies hold more than 30 pairs (Cramp 1988). In much of the suitable habitat in Europe, the nesting density of House Martins is typically around one to two pairs per km^2^ (Cramp 1988), so Stora Karlsö (an island of 2.5 km^2^ with 170 pairs of House Martins in 2013) represents an unusually high breeding density for this species. House Martin numbers in the lighthouse colony increased from 23 pairs in 1984 to 41 in 1998, 51 in 2005 and 150 in 2013 (Hedgren & Kolehmainen 2006, Länsstyrelsen 2006). This increase contrasts with an estimated 30–49% decline in House Martins throughout Sweden over a 30-year period (Ottvall *et al*. 2009). Colony-specific population parameters of Common Guillemots, including adult survival (Österblom *et al*. 2004) and breeding success (Kadin *et al*. 2012), are consistently high, and indicate, together with census counts at Stora Karlsö (Hedgren & Kolehmainen 2006), a substantial increase in the Common Guillemot population. Counts of Razorbills, the other large population of sprat-feeding seabirds on Stora Karlsö, also show a marked increase during recent years (Länsstyrelsen 2006). Previous studies indicate strong links between the dynamics of several Common Guillemot population parameters and the dynamics of the Sprat stock (Österblom *et al*. 2006, Kadin *et al*. 2012). It is likely that the growth of the Guillemot population has been enabled by a dramatic increase in the Baltic Sea Sprat stock, in turn affected by overfishing of its main predator Cod *Gadus morhua* and changing climatic conditions (Casini *et al*. 2008, 2009). Overfishing of Cod can have profound effects on entire marine food webs – examples include effects on pelagic fish stocks, zooplankton, phytoplankton and nutrients in the Scotian Shelf (Frank *et al*. 2005), and potentially also phytoplankton biomass in the Baltic Sea (Casini *et al*. 2009). We speculate that the increasing numbers in the House Martin colony on Stora Karlsö may be a consequence of the large and increasing Common Guillemot population (in turn substantially affected by marine ecosystem dynamics), through the provision of an abundant coastal insect prey subsidy derived from nutrient run-off into coastal waters from the adjacent seabird colony.

The δ^15^N and especially the δ^13^C values of House Martin nestling feathers indicate that seabirds play an important role in the transfer of nutrients from the marine environment to this local population. Other island populations of insectivorous birds may also be influenced by the presence of seabirds. For example, the St Kilda Wren *Troglodytes troglodytes hirtensis* (Miles 2011) and the Fair Isle Wren *Troglodytes troglodytes fridariensis* (Aspinall & Aspinall 2011) occur at remarkably high local densities, especially on the sea cliffs (Forrester & Andrews 2007), which hold internationally important populations of seabirds. Their numbers may be dependent on nutrient inputs to these islands from seabirds. There is thus a need to understand better the degree of dependence of other passerine species and populations on seabird subsidies, in particular in the context of declining seabird populations (Caut *et al*. 2012). We argue that such cross-scale dynamics represent an interesting challenge for agencies defining their management mandate by traditional ecosystem boundaries.

### 

A.C. was supported by a BBSRC studentship and the National Trust for Scotland. We thank Aron Hejdström and Johan Träff for helping to collect feather samples, and Ola Wizén for House Martin survey data. We also thank Håkan Elmqvist and Yngve Brodén for their expert advice on chironomids. J.H.S. and H.Ö. were supported by MISTRA, WWF Sweden and the Baltic Ecosystem Adaptive Management Program. Support from the Karlsö Jagt och Djurskyddsförening was much appreciated.
